# Preparation and Performance of Antibacterial Polyvinyl Alcohol/Polyethylene Glycol/Chitosan Hydrogels Containing Silver Chloride Nanoparticles via One-step Method

**DOI:** 10.3390/nano9070972

**Published:** 2019-07-03

**Authors:** Gang Li, Daohai Zhang, Shuhao Qin

**Affiliations:** 1 College of Materials Science and Metallurgy Engineering, Guizhou University, Guiyang 550025, China; 2 National Engineering Research Center for Compounding and Modification of Polymeric Materials, National and Local Joint Engineering Research Center for Functional Polymer Membrane Materials and Membrane Processes, Guiyang 550014, China

**Keywords:** silver chloride nanoparticles, hydrogels, mechanical properties, antibacterial activity

## Abstract

Silver nano-particles (AgNPs)-filled antibacterial materials have been widely employed in the fields of biology and biomedicine. However, AgNPs have shown obvious cytotoxicity. Hence it is more reasonable to use silver chloride nanoparticles (AgCl NPs) to prepare antibacterial materials due to the slow release of silver ions created by AgCl NPs formed in the chitosan. In this experiment, a useful antibacterial hydrogel for skin repairation was prepared by exploring the relationship between AgCl NPs and cytotoxicity. It is worth noting that the crosslinked network structure was successfully obtained in an antibacterial AgCl/PVA (Polyvinyl alcohol)/PEG (Polyethylene glycol)/CS (Chitosan) hydrogel materials by the hydrothermal method. In detail, the dynamic particle size distribution of AgCl NPs was relatively uniform, which is analyzed by a dynamic light scattering (DLS). The internal structure of the lyophilized hydrogel showed obvious porous structure, indicating that the hydrogel had high water content. The result of X-ray photoelectron spectroscopy (XPS) confirmed the existence of a silver element. The release concentration of silver ions was analyzed by inductively coupled plasma (ICP) to study the effect of silver ions release concentration on the antibacterial activity and cytotoxicity of hydrogel. The results show that the lower concentration of silver ions can make the hydrogel have good antibacterial activity and low cytotoxicity. The bacteriostatic rate of the antibacterial hydrogel was over 90%. Simultaneously, the mechanical properties test shows that the hydrogel has good mechanical properties, which can be widely used as an antibacterial material.

## 1. Introduction

Skin burns or extensive damage is usually easy to be infected, causing the wound to be difficult to heal during the process of treatment [1]. Therefore, it is necessary to provide a good environment for the cells to adhere and grow targeting at rapidly repairing damaged skin, further inhibiting the growth of bacteria. Usefully, the hydrogel can absorb body fluids and exchange the substances with the external environment. Simultaneously, the hydrogel can be availed as a carrier for the drug, in terms of the sustained release treating the wound [2]. Here, AgNPs and their compounds are often employed as antibacterial agents because of their broad-spectrum antibacterial ability. However, released silver ions by AgNPs will damage the cell membrane of bacteria, inhibit protein synthesis, and cause the death of the bacterial. Mainly, the bacteria cannot pass through proliferation and differentiation, resulting in infection of the body [3,4,5]. Therefore, antibacterial materials can be prepared by using hydrogel as a carrier and loaded with AgNPs and its compounds as artificial skin for skin repair [6,7].

Hydrogels can be used as artificial skin for the repair of damaged skin. Due to the full contact with the skin, it therefore needs to have good biocompatibility and bioactivity, such as polyvinyl alcohol (PVA), polyethylene glycol (PEG), and Chitosan (CS) [8,9]. As is well-known, medical grade PVA has high safety, low toxicity, good biocompatibility, and stable chemical properties, which is widely used in medicine. These features encourage the extensive research and focus on PVA-based materials for a variety of industrial and medical manufacturing, including wound dressings, cartilage repair, and drug delivery. In addition, it has a large amount of hydroxyl groups, which can form a hydrogel with good mechanical properties by physical action [10,11]. Usually, the hydrogel prepared by PVA has poor mechanical properties. And it can be improved by blending with other polymers to construct the physical cross-linking network of the hydrogel. PEG has good water soluble due to an abundance of hydroxyl groups at both ends of the molecular chain. Thus, it can be well compatible with many polar substances, especially for CS [12]. CS has good biocompatibility, bioactivity, biosafety, and biodegradability [13], and has a wide range of applications in medicine, food, chemical, biomedical engineering, and other fields [14,15,16]. CS has a large amount of free amino groups and is easily cationized under acidic conditions, thereby inhibiting the growth of bacteria [17]. Therefore, CS can be used to prepare an antibacterial hydrogel for good mechanical properties [18].

The use of antibiotics tends to resist the bacteria. For example, AgNPs and their compounds have a broad spectrum of antibacterial ability [19]. Therefore, inorganic particles of AgNPs are frequently added to hydrogels with the goal of preparing antibacterial materials [20]. Interestingly, when AgNPs are used as antibacterial agents to manufacture an antibacterial hydrogel, the release concentration of silver ions can be controlled by modifying AgNPs [21]. High concentrations of silver ions have strong cytotoxicity to cells. While the low concentration of silver ions below the threshold of 10 ppm, the antibacterial hydrogel loaded with AgNPs exhibits a significant antibacterial effect as improving cell proliferation [22]. In this work, we explored the use of AgCl NPs as an antibacterial agent for the preparation of antibacterial hydrogels. Silver ions combined with chloride ions to form AgCl NPs precipitatation [23]. The presence of chloride ions can reduce the release concentration of silver ions, which leading to the relatively weak cytotoxicity of AgCl NPs. By adjusting the content of AgCl NPs to reduce the cytotoxicity of the hydrogel, an antibacterial hydrogel is simultaneously capable of achieving antibacterial and promoting cell adhesion and proliferation [24]. This method provides a meaningful reference for the preparation of antimicrobial materials and the reparation of damaged skin [25].

## 2. Materials and Methods

### 2.1. Materials

These experimental materials were purchased and used in the preparation of hydrogels. Polyvinyl alcohol (PVA) was purchased from Tianjin Fuyu Chemical Co., Ltd. (Tianjin, China). Chitosan (CS) was purchased from Shanghai Maclean Biochemical Technology Co., Ltd. (Shanghai, China). Polyethylene glycol (PEG) and Silver nitrate (AgNO_3_) were purchased from Sinopharm Chemical Reagent Co., Ltd. (Shanghai, China). Hydrochloric acid (HCl) was purchased from Chongqing Chuandong Chemical Co., Ltd. (Chongqing, China). Deionized water was homemade. Mouse embryonic fibroblast cells (NIH3T3) were purchased from Shanghai Zhongqiao Xinzhou Biotechnology Co., Ltd. (Shanghai, China). Fetal bovine serum (FBS) was purchased from Bovogen Biologicals Pty Ltd. (Melbourne, Australia). Trypsin-EDTA and medium were purchased from Gibco Corporation (Grand Island, NY, USA). Phosphate Buffered Saline (PBS) was purchased from Boster Biological Technology co., Ltd. (Wuhan, China). Additionally, cell Counting Kit-8 (CCK-8) was purchased from MedChem Express LLC (NJ, USA).

### 2.2. Preparation of AgCl NPs

Firstly, CS was dissolved in dilute HCl to configure 50 mL of a 1 wt % CS solution. Meaningfully, the CS solution contains a large amount of chloride ions, which can be combined with silver ions to form AgCl NPs. Therefore, 0 g, 1.062 g, 2.123 g, and 3.185 g of AgNO_3_ were dissolved in 200 ml of water, respectively. Then, 50 ml of the CS solution was added into 200 ml AgNO_3_ solution. And further the silver ions in the solution consumed chloride ions to form stable solution containing AgCl NPs, ready for hydrogel preparation [26]. The particle size and distribution range of the AgCl NPs were performed on a laser light scattering system (Zetasizer Nano-ZS90, Great Malven, England, UK).

### 2.3. Preparation of Antibacterial Hydrogel

**Figure 1:**
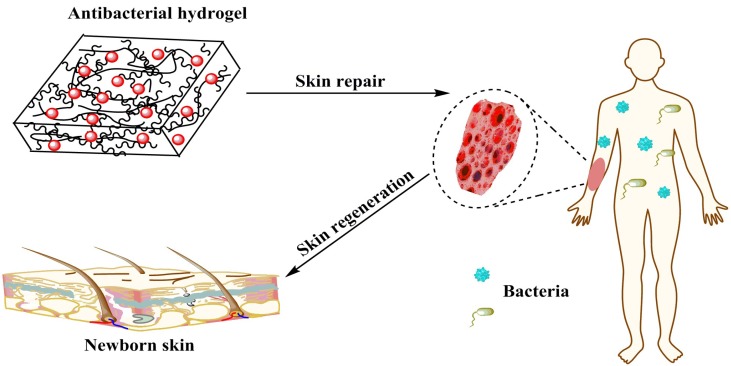
Antibacterial hydrogel for skin repair.

The antibacterial hydrogel was prepared by a hydrothermal method, and the contents of the components of the hydrogel are shown in Table 1. About 5.0 g of PVA and 5.0 g of PEG were weighted into the beaker with water (50 mL), and then were heated into 100 ℃ for fully dissolving the PVA under magnetic stirring. After the PVA was completely dissolved, 20 ml of the solution taken from 250 mL of the CS solution containing AgCl NPs, was added into a mixed solution of PVA and PEG. After being thoroughly mixed, the surface film layer of the cooling solution was removed and cast into a surface dish. After removing the bubbles, the solution is placed in a refrigerator and freeze-thawed at −40 °C for 4 times to form a crosslinked structure by physical action inside the hydrogel in order to obtain an antibacterial hydrogel with a stable structure. Subsequently, the hydrogel is immersed in water for a long time until removal of part of the NO_3_^−^ by ion. Finally only small amount of NO_3_^−^ ions was obtained in hydrogel. As shown in Figure 1, the antibacterial hydrogel obviously repairs the damaged skin, inhibits bacterial growth during the repair process, avoids infection, and ultimately achieves skin regeneration.

### 2.4. Lyophilization of Hydrogel

The freeze dryer was turned on to pre-cool well for 2 h, while the hydrogel was frozen at −40 °C. The frozen hydrogel was placed in a cold well, and the vacuum pump was turned on for lyophilization for 48 h to obtain a lyophilized hydrogel in which the morphology remained intact. The freeze-dried hydrogel was used for scanning electron microscope (Quanta FEG-250, FEI, Hillsboro, OR, USA) observation of the cross-sectional structure of the hydrogel. The elements in the sample were analyzed using an energy dispersive spectrometer and X-ray energy spectrum analysis (K-Alpha+, TMO, Waltham, MA, USA). The presence and crystal structure of the AgCl NPs in the freeze-dried hydrogel were analyzed by X-ray diffractometry (D8 Rigaku9000, BrukerAXS, Karlsruhe, Germany). The freeze-dried hydrogel was subjected to synchronous thermal analysis by a thermogravimetry-differential thermal synchronous analyzer (STA449F3, NETZSCH, Selb, Germany). The release concentration of silver ions is analyzed by inductively coupled plasma (Agilent 720ES, Agilent, CA, USA).

### 2.5. Mechanical Properties of Hydrogels

Hydrogels should have excellent mechanical properties when it is used as antimicrobial material. Therefore, the mechanical properties of the hydrogel were tested using a microcomputer-controlled universal testing machine (CMT 6104, Mest Industrial Systems Co., Ltd., Shanghai, China) in accordance with the GB/T 1040.3-2006 standard. The hydrogel was prepared to meet the standard test splines for mechanical testing, and the solution was cast into the prepared standard spline mold and subjected to multiple freeze-thaw cycles to obtain standard splines.

### 2.6. In Vitro Antibacterial Activity of the Hydrogel

The liquid medium bacteriostatic test is used to measure the optical density (OD) value of the medium containing hydrogel. The two cells (*E.coli* DH5α, *S.aureus* CMCC26003) were cultured in Luria-Bertani (LB) medium at 220 rpm and 37 °C until the OD600 was about 0.6, corresponding to a concentration of 10^8^ CFU/mL, diluted 1:1000. The ratio was diluted to a final concentration of 10^5^ CFU/mL, and the samples to be tested were added to 2 mL of the bacterial solution (three parallel replicates were set for each group), and cultured at 37 °C, 220 rpm for 72 h. The OD value is detected.

### 2.7. In Vitro Cell Culture

The hydrogel was sterilized under ultraviolet light for 30 min. NIH3T3 was used to perform cell culture experiments to verify the cell cytotoxicity of hydrogels that can be used for skin repair [27]. Clip the sterilized material into a 24-well plate, collect the log phase of NIH3T3 cells, perform cell counting, adjust the cell suspension concentration to 1 × 10^4^ cells/ml, and place in a confocal culture dish to make the cells to be tested to 1 × 10^4^ cells/well, 1 ml per well, allowing cells and materials to be incubated together and incubated for 24 h in a 5% CO_2_, 37 °C cell culture incubator. After the incubation, the cells were washed twice with PBS and fixed with 4% paraformaldehyde for 30 min. The residual paraformaldehyde was washed away with PBS. And FITC-Phalloidin was added in the dark for 30 min. After staining, wash three times with PBS, aspirate PBS, add immunofluorescent seals with DAPI, and after 10 min, observe and photograph with a Laser scanning confocal microscope (TCS SP8, LEICA, Solms, Germany). FITC-Phalloidin uses an excitation wavelength of 488 nm and an emission wavelength range of 500–540 nm. DAPI uses an excitation wavelength of 405 nm and an emission wavelength range of 430–460 nm. Take a picture with a 630X oil mirror [28].

## 3. Results and Discussion

**Figure 2:**
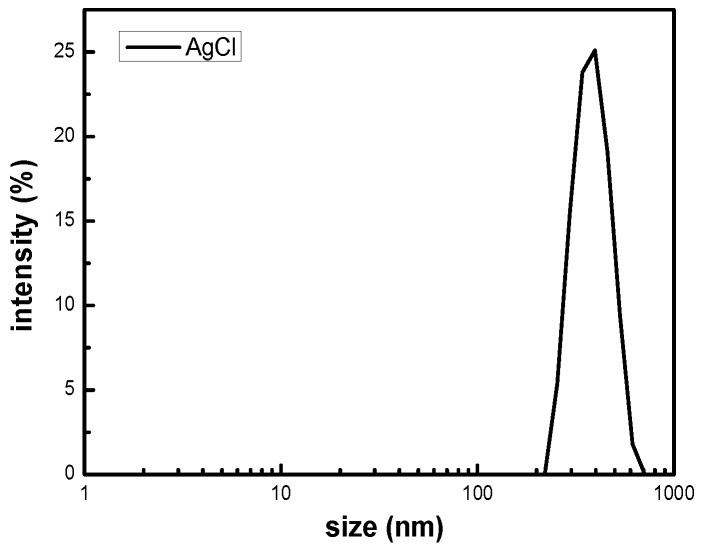
Dynamic particle size distribution of AgCl NPs.

The size distribution of the silver chloride was studied by dynamic light scattering in the solution. It can be seen from the Figure 2 that the particle size of silver chloride is mainly distributed in the range from 295.3 to 458.7 nm, which has a relatively narrow particle size distribution. The results of DLS consistently disclose that the polydispersity coefficient (PDI) of the silver chloride particles in solution was 0.296, exhibiting a uniform size with an average hydrodynamic diameter of 454.1 nm. All of the silver chloride particles in the solution demonstrate the nanometer size. Foreseeably, AgCl NPs can be used to prepare hydrogels having antibacterial activity [29].

**Figure 3:**
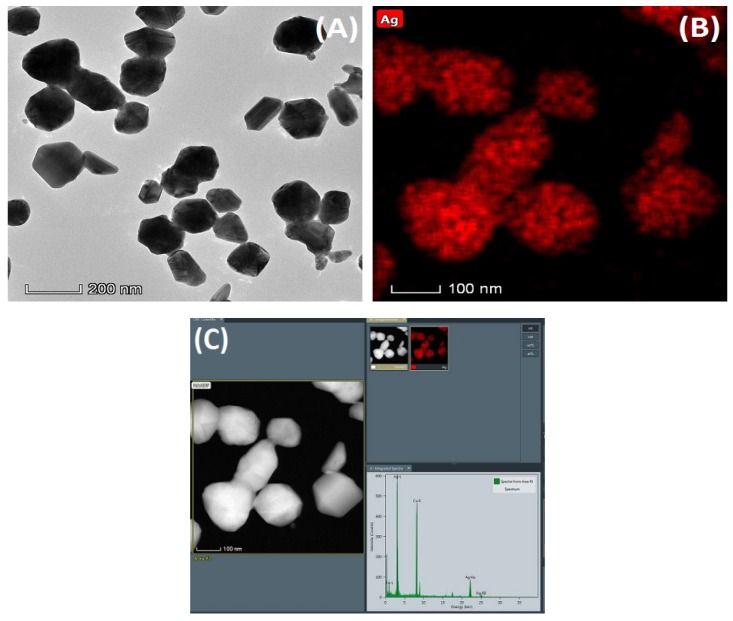
Morphological observation and elemental analysis of AgCl NPs by transmission electron microscopy. (**A**) Nanoparticle morphology, (**B**) Silver element mapping, (**C**) Silver element content analysis

The morphology observation and elemental analysis of the nanoparticles were carried out by transmission electron microscopy, as shown in Figure 3. Figure 3A is a topographical view of the nanoparticles. And Figure 3B,C are elemental analysis diagrams of the nanoparticles. From the images, it can be found that the size of the nanoparticles is relatively uniform. In addition, elemental analysis of the nanoparticles confirmed that these nanoparticles contained a large amount of silver element.

**Figure 4:**
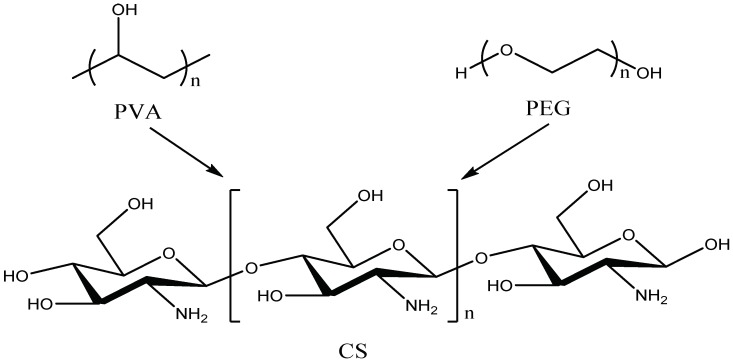
The figure shows the molecular formulas of PVA, PEG, and CS, respectively.

**Figure 5:**
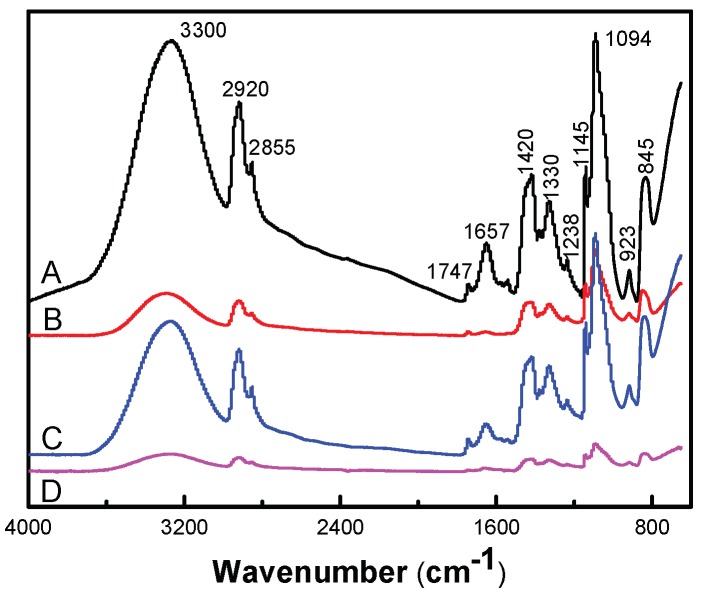
FT-IR (Fourier transform infrared spectroscopy) of AgCl/PVA/PEG/CS hydrogels loaded with (**A**) 0 mM, (**B**) 2.5 mM, (**C**) 5 mM, and (**D**) 7.5 mM AgCl NPs.

Figure 4 shows the molecular formulas of PVA, PEG and CS, respectively. The PVA molecular chain contains a large number of hydroxyl groups. Additionally, the molecular chain of CS contains a large amount of hydroxyl groups and amino groups But only two hydroxyl groups are contained in the molecular chain of PEG. When PVA, PEG, and CS are blended to prepare a hydrogel, an intermolecular interaction force between the molecular chains will be formed due to the hydroxyl group and the amino group, leading to a physical crosslinked network structure in hydrogen. Figure 5 presented an FT-IR spectra of antimicrobial hydrogel containing various amounts of AgCl NPs. The chemical bond and functional group information of AgCl/PVA/PEG/CS hydrogel were obtained by FT-IR measurement. Additionally, the chemical composition of the hydrogel and possible chemical reactions were analyzed in detail. For instance, the characteristic broad band around the 923 and 1145 cm^−1^ peaks corresponds to the saccharin structure of CS. The characteristic peaks at 1657 cm^−1^ are absorption peaks of –(NH_2_) and –(OC–NH_2_). Characteristic absorption peaks around 3300 cm^−1^ correspond to –(OH) and –(NH) tensile vibrations [30]. Many absorption peaks at 2920, 1420, 1330, 1094 and 845 cm^−1^ in the infrared spectrum are attributed to –(CH_2_), –(CH–OH), –(CH–OH), –(C–O) and –(C–C) resonance of PVA [31]. The characteristic absorption peaks at 845 and 1420 cm^−1^ correspond to the stretching vibrations of C–O–C and C–C of PEG, respectively. PVA, CS and PEG were confirmed to be components of the hydrogel by infrared spectroscopy [32].

**Figure 6:**
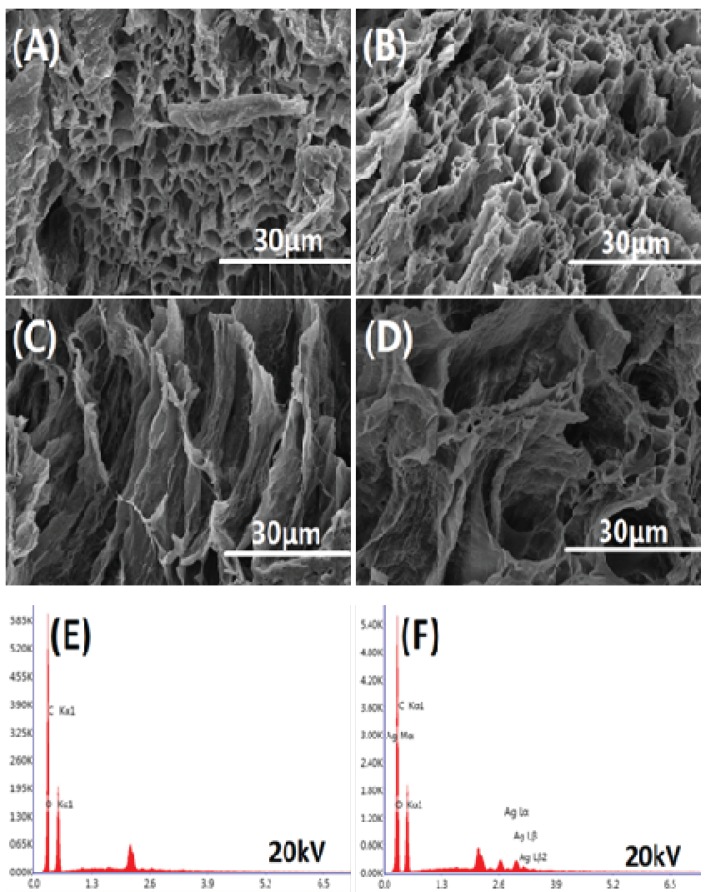
The cross-sectional morphology of hydrogels are (**A**) AS0, (**B**) AS25, (**C**) AS50, and (**D**) AS75; EDS (Energy Dispersive Spectrometer) element analysis of hydrogels are (**E**) AS0 and (**F**) AS75.

Figure 6 shows the cross-sectional morphology and EDS elemental analysis of the freeze-dried hydrogel. The freeze-dried hydrogel has a porous structure inside, indicating that the hydrogel contains a large amount of water. The internal porous structure is mostly tubular structures stacked on each other, which facilitates the transport of moisture. Simultaneously, EDS elemental analysis was performed on AS0 and AS75. It can be seen from Figure 6E,F that the hydrogel AS75 contains silver element. Moreover, Table 2 shows that the hydrogel AS75 has a silver element content of 1.82 wt %, and the presence of silver as an antibacterial agent allows the hydrogel to have antibacterial activity.

**Figure 7:**
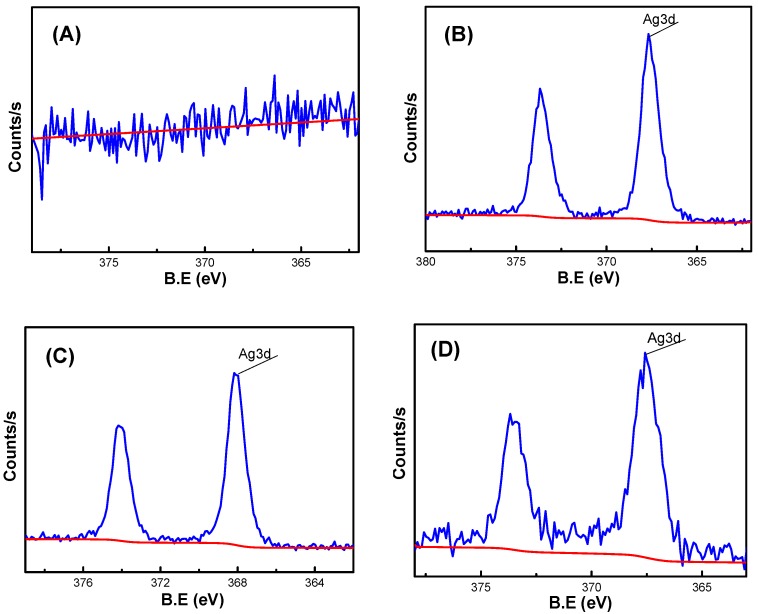
XPS analysis of the composition of the hydrogel, (**A**) AS0, (**B**) AS25, (**C)** AS50, (**D**) AS75

As shown in Figure 7, XPS analysis was performed on hydrogels AS0, AS25, AS50 and AS75. From the Analysis of the electron spectroscopy, it showed that the hydrogel AS0 hardly contain silver element, while the hydrogels AS25, AS50, and AS75 contain a small amount of silver element, which corresponds to the EDS elemental analysis of the hydrogel, demonstrating the presence of silver element in the hydrogel. Table 3 shows the contents of various elements in the hydrogel, which mainly analyzed the change of the content of silver element in the hydrogel. The content of silver element in the hydrogel firstly increases and then decreases. However, the content of silver in the hydrogel should show an increasing trend, which indicates that the test results of the hydrogel AS75 have some errors. Simultaneously, the EDS data can be used to demonstrate that the hydrogel AS75 has a higher silver content than the hydrogel AS50, and its silver content is 1.82 wt %.

**Figure 8:**
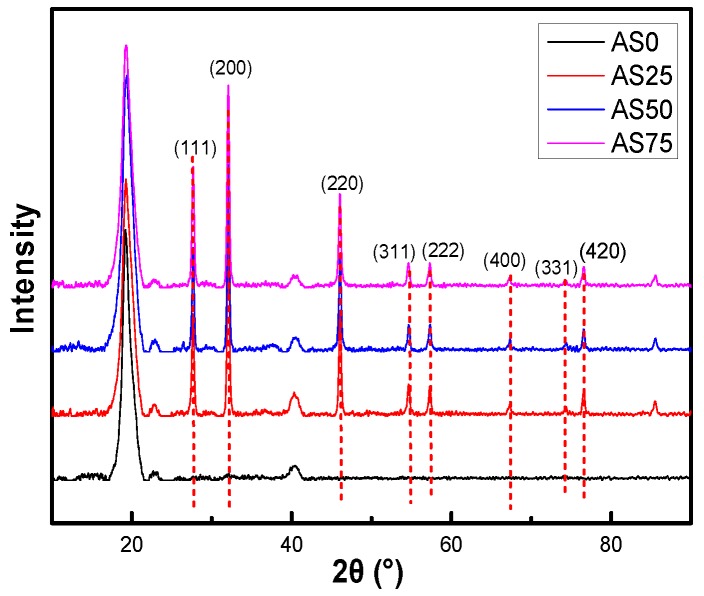
XRD (X-ray diffraction ) pattern of Hydrogels.

The crystalline properties of AgCl NPs in the hydrogel were investigated by XRD pattern, as shown in Figure 8. Diffraction peaks observed in the 2θ range of 20° to 90° in the XRD spectrum of AgCl NPs were 27.63°, 32.02°, 46.00°, 54.69°, 57.36°, 67.16°, 74.41°, and 76.67°. They are corresponding to the face-centered cubic structures (111), (200), (220), (311), (222), (400), (331), and (420) planes of AgCl NPs, respectively [33,34]. This indicates the addition of silver nitrate to the chitosan solution, which consumes chloride ions to form AgCl NPs. A CS solution containing AgCl NPs was introduced during the preparation of the hydrogel to obtain a hydrogel containing the antibacterial inorganic particles AgCl NPs [35]. 

**Figure 9:**
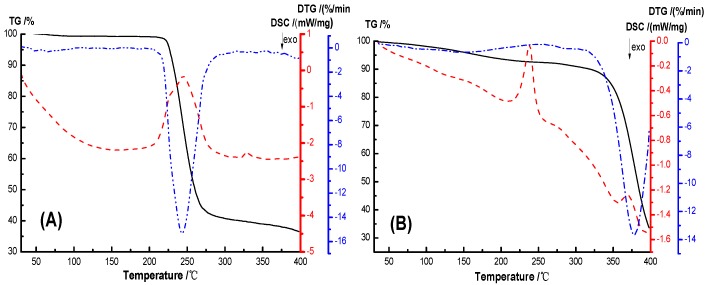

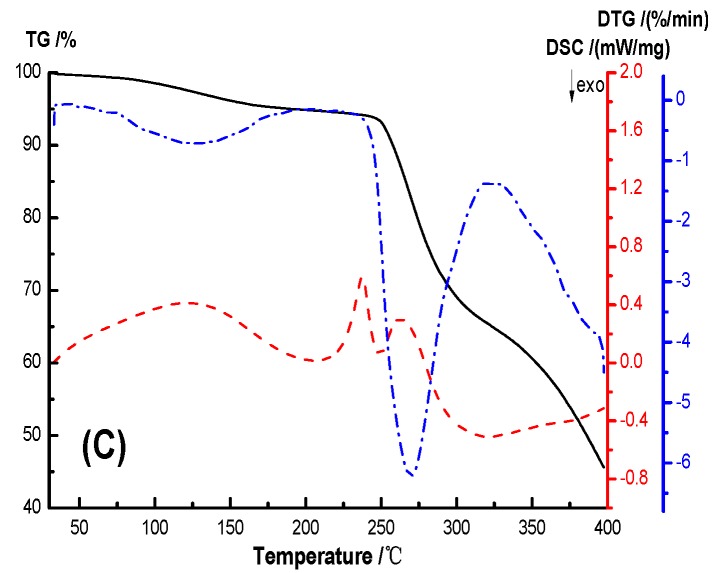
TG-DSC (Thermogravimetric differential thermal analyze) curves of Hydrogels, (**A**) PVA, (**B**) AS0, and (**C**) AS50.

The thermal stability and crystallization properties of the hydrogel were analyzed by TG-DSC, as shown in Figure 9. In the TG-DSC curves, the initial thermal weight loss temperature T_5%_ of PVA, AS0, AS50 are 226.45 °C, 161.77 °C, 177.14 °C, and the initial thermal weight loss temperature T_10wt%_ are 231.10 °C, 315.84 °C, 258.23 °C, respectively, and the temperature difference between them are 4.65 °C, 153.87 °C, 81.09 °C, respectively. The temperatures of the maximum weight loss rate of PVA, AS0 and AS50 are 243.3 °C, 378.2 °C, 269.7 °C, respectively. This indicates that the hydrogel has a higher thermal stability than PVA. The initial thermogravimetric temperature of the hydrogel is lower than PVA because the PEG used to prepare the hydrogel has a molecular weight of 400 and thermal degradation occurs at lower temperatures. AS0’s initial thermal weight loss temperature and maximum thermal weight loss rate temperature are higher than AS50, which may be due to the good thermal conductivity of AgCl NPs, accelerating the thermal degradation of hydrogel. The crystallization peak areas of PVA, AS0, AS50 are 609.84 J/g, 57.04 J/g, 101.34 J/g, respectively, and the crystallinity of PVA, AS0, and AS50 was known. This is mainly ascribed to hydrogen bonds formed between molecules in the hydrogel, which reduces the mobility of the molecular chain. And thus the crystallinity of the material is lowered. Simultaneously, AgCl NP acts as a nucleating agent to promote the hydrogel AS50 crystallization [36].

**Figure 10:**
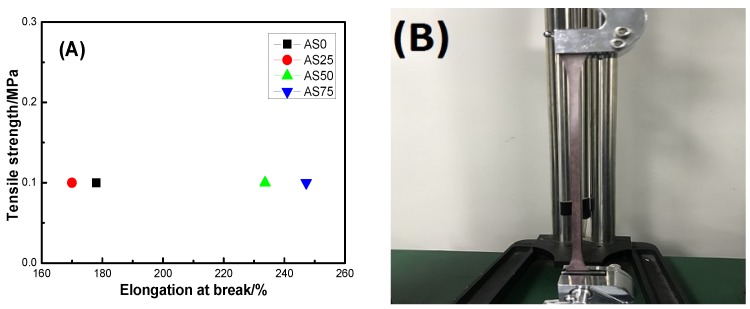

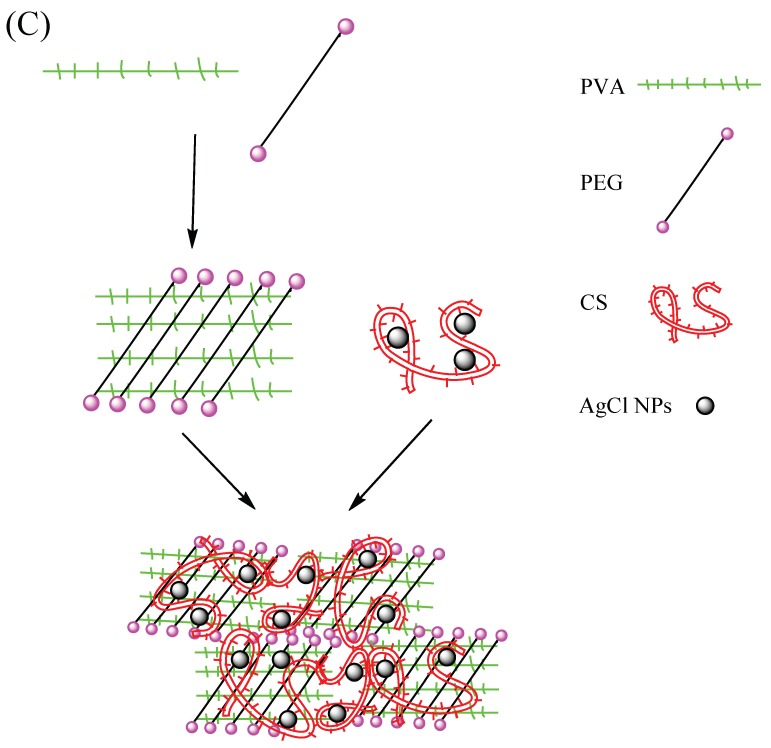
(**A**) Elongation at break and tensile strength of hydrogel, (**B**) schematic diagram of the stretching process of hydrogel AS25, and (**C**) schematic diagram of the formation of a hydrogel containing AgCl NPs having a physically crosslinked network structure.

The elongation at break and tensile strength of the hydrogel are shown in Figure 10. In detail, Figure 10A is the elongation at break and tensile strength of the hydrogel. Figure 10B showed the stretching process of the hydrogel AS25. And Figure 10C demonstrated the formation of a hydrogel having excellent mechanical properties. Firstly, the spline mold is prepared according to the GB/T 1040.3-2006 standard, and the spline is obtained by casting molding and repeated freeze-thaw treatment. The mechanical properties of the hydrogel were analyzed by a microcomputer-controlled universal testing machine. From Figure 10C, there are only a small number of hydroxyl groups on both ends of the PEG molecular chain, the intermolecular force between PVA and PEG is weak, resulting in the poor mechanical properties. After the introduction of CS, due to the presence of a large number of hydroxyl groups and amino groups on the CS molecular chain, the intermolecular forces formed the strong hydrogel. And at the same time, the presence of AgCl NPs causes the formation of the physical entanglement point between polymer molecules. As can be seen from the figure, the tensile strength of the hydrogel is 0.1 MPa, indicating that the hydrogel has a certain mechanical strength and can be used as an antibacterial material such as artificial skin. The hydrogel has an elongation at break between 170% and 250% and a maximum elongation at break of 247.2%, indicating that the hydrogel has good elastic behavior. The elongation at break of hydrogel AS0 is greater than the elongation at the break of hydrogel AS25, which may be caused by the uneven distribution of AgCl NPs inside hydrogel AS25. The good mechanical properties of hydrogels create conditions for hydrogels to be used as antibacterial materials for artificial skin [37].

Table 4 shows the release concentration of silver ions in the solution after the hydrogel was immersed in water for 24 h. From Table 4, the silver ions release concentrations of hydrogels for AS0, AS25, AS50, AS75 were below 0.02 mg/L, 0.041 mg/L, 2.762 mg/L, and 3.264 mg/L, respectively. The release concentration of silver ions trends to increase gradually. Interestingly, the release concentration of silver ions of the hydrogel AS0 is almost zero. This corresponds to the amount of added AgCl NPs in the experiment. Simultaneously, according to the release concentration of silver ions, the relationship between the release concentration of silver ions and antibacterial activity, and the release concentration of silver ions on cell proliferation were investigated.

**Figure 11:**
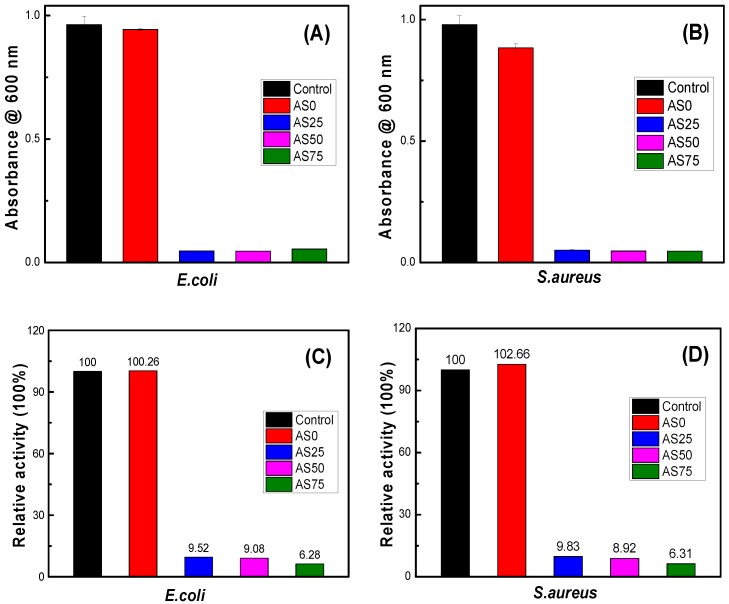
Quantitative analysis of proliferation of E.coli (**A**) and S.aureus (**B**), and the relative activity of E.coli (**C**) and S.aureus (**D**), culture in LB medium containing four groups of hydrogels.

Figure 11 is an antibacterial experiment modeled according to *E.coli* and *S.aureus*. It can be seen from the figure that there is no significant difference in absorbance between hydrogel PVA/PEG/CS and blank control, indicating that hydrogel PVA/PEG/CS has no bacteriostatic effect. When the hydrogel contains AgCl NPs, the absorbance of hydrogel AgCl/PVA/PEG/CS is significantly reduced, showing significant bacteriostatic effect. As can be seen from Figure 11C,D, the absorbance of hydrogels AgCl/PVA/PEG/CS are below 10. This indicates that the antibacterial hydrogel has a bacteriostatic rate of more than 90% and exhibits excellent antibacterial activity. Table 4 shows the release concentration of silver ions of the hydrogel. The experimental results confirm that the change of AgCl NPs content in hydrogel has little effect on the antibacterial effect of hydrogel. Therefore, the content of AgCl NPs can be adjusted to reduce the cytotoxicity of the antibacterial hydrogel without affecting the antibacterial effect.

**Figure 12:**
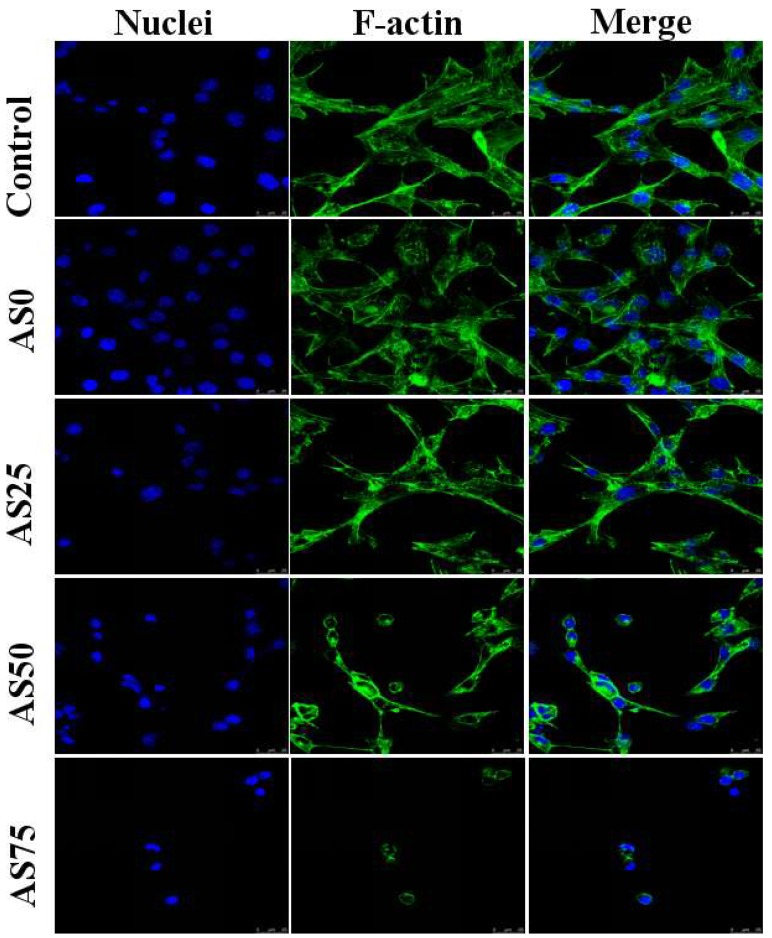
CLSM images of NIH3T3 cells cultured for 24 h of four groups of hydrogels (AS0, AS25, AS50, AS75) showing nucleus stained with DAPI (blue) and F-actin stained with FITC-phalloidin (green).

NIH3T3 cells were cultured in a medium containing hydrogels and the morphology of the cells was observed by confocal microscopy, as shown in Figure 12. By observing the morphology of the cells in the figure, it can be found that the hydrogel containing no AgCl NPs has a good promoting effect on the growth of cells. And thus can be used as an artificial skin. With the increase of the content of AgCl NPs, the growth of NIH3T3 cells was gradually inhibited. As can be seen from the figure, the cells were cultured in a medium containing hydrogel AS75, and cell growth was significantly inhibited. As shown in Table 4, the content of AgCl NPs in the hydrogel AS75 is higher, leading to the higher concentration of released silver ions, caused cell death due to the large cytotoxicity. When the content of AgCl NPs in the hydrogel is low, it shows weak cytotoxicity. Therefore, the content of AgCl NPs below the antibacterial threshold can reduce the cytotoxicity of the hydrogel, which is availed to the reparation of the damaged skin. For the purpose of antibacterial as well as inhibiting the adhesion and proliferation of cell-like organisms, hydrogel AS75 can be used as a benchmark to achieve the dual role of antibacterial and the inhibition of cell-based bio-adhesion [38,39].

## 4. Conclusions

The hydrogel AgCl/PVA/PEG/CS was successfully prepared by hydrothermal method, which is relatively simple and green. The physical crosslinked network structure is formed between the molecular chains of PVA, PEG, and CS by blending, thereby imparting good mechanical properties to the hydrogel to meet the application requirements for skin repair. The antibacterial agent of AgCl NPs is introduced into the hydrogel to impart a certain antibacterial ability for hydrogel. The antibacterial experiment results showed that the bacteriostatic rate of the antibacterial hydrogel was over 90%, indicating that the antibacterial hydrogel has excellent antibacterial activity. Additionally, it can inhibit the growth of *E.coli* and *S.aureus*. Simultaneously, cytotoxicity experiments show that antibacterial hydrogels containing AgCl NPs have certain cytotoxicity to cells. Reducing the content of AgCl NP can decrease the release concentration of silver ions and thus weaken the cytotoxicity of hydrogels. Thus, it can be used as artificial skin to guide skin regeneration.

## Figures and Tables

**Table 1:** The composition of the antibacterial hydrogel.

Sample	PVA/g	PEG/g	CS/g	Ag/mg
AS0	5	5	0.04	0
AS25	5	5	0.04	54.02
AS50	5	5	0.04	107.98
AS75	5	5	0.04	162.00

**Table 2:** Analysis of the content of elements in hydrogel by EDS.

Sample	C	O	Ag
AS0	60.85	39.15	0
AS75	56.47	41.71	1.82

**Table 3:** Analysis of the content of elements in hydrogel by XPS.

Sample	C	N	O	Ag
AS0	67.58	0.56	31.86	0
AS25	66.03	1.05	32.49	0.43
AS50	66.1	1.43	31.64	0.83
AS75	75	1.26	23.52	0.22

**Table 4:** Analysis of silver ion release concentration of hydrogel in water by ICP (Inductively coupled plasma).

Sample	Test Element	Test Solution Element Content (mg/L)	Dilution Factor	Sample Element Content (mg/L)
AS0	Ag	<0.02	1	<0.02
AS25	Ag	0.041	1	0.041
AS50	Ag	2.762	1	2.762
AS75	Ag	3.246	1	3.246
